# Orthopedic surgeons’ and neurologists’ attitudes towards second opinions in the Israeli healthcare system: a qualitative study

**DOI:** 10.1186/2045-4015-1-30

**Published:** 2012-07-24

**Authors:** Geva Greenfield, Joseph S Pliskin, Shlomo Wientroub, Nadav Davidovitch

**Affiliations:** 1 Department of Primary Care and Public Health, School of Public Health, Imperial College London, The Reynolds Building, St Dunstans Road, London, W6 8RP, UK; 2 Department of Health Systems Management, Ben-Gurion University of the Negev, P.O. Box 653, Beer-Sheva, 84105, Israel; 3 Department of Industrial Engineering and Management, Ben-Gurion University of the Negev, Beer-Sheva, 84105, Israel; 4 Department of Pediatric Orthopedics, Dana Children’s Hospital, Tel-Aviv Sourasky Medical Centre, Sackler Faculty of Medicine, Tel-Aviv University, Tel-Aviv, 69978, Israel

**Keywords:** Second opinion, Health policy, Public medicine, Private medicine, Inequalities, Qualitative research

## Abstract

**Background:**

Second opinion is a treatment ratification tool that may critically influence diagnosis, treatment, and prognosis. Second opinions constitute one of the largest expenditures of the supplementary health insurance programs provided by the Israeli health funds. The scarcity of data on physicians’ attitudes toward second opinion motivated this study to explore those attitudes within the Israeli healthcare system.

**Methods:**

We interviewed 35 orthopedic surgeons and neurologists in Israel and qualitatively analyzed the data using the Grounded Theory approach.

**Results:**

As a common tool, second opinion reflects the broader context of the Israeli healthcare system, specifically tensions associated with health inequalities. We identified four issues: (1) inequalities between central and peripheral regions of Israel; (2) inequalities between private and public settings; (3) implementation gap between the right to a second opinion and whether it is covered by the National Health Insurance Law; and (4) tension between the authorities of physicians and religious leaders. The physicians mentioned that better mechanisms should be implemented for guiding patients to an appropriate consultant for a second opinion and for making an informed choice between the two opinions.

**Conclusions:**

While all the physicians agreed on the importance of the second opinion as a tool, they raised concerns about the way it is provided and utilized. To be optimally implemented, second opinion should be institutionalized and regulated. The National Health Insurance Law should strive to provide the mechanisms to access second opinion as stipulated in the Patient’s Rights Law. Further studies are needed to assess the patients' perspectives.

## Background

Second opinion is a treatment ratification tool that can critically influence diagnosis, treatment, and prognosis. Mandatory second opinion programs were introduced in the United States in the 1970s as a pre-authorization tool before elective surgery [1]. Indeed, utilization review programs substantially reduced the number of diagnostic and surgical procedures [2,3] and provided evidence for the potential of second opinions to contribute to treatment optimization and the reduction of unnecessary risks and costs. Further studies have shown major discrepancies between first and second opinions [4-6]. Recent decades have seen the second opinion evolve from a pre-authorization tool to become a patient’s right [7]. Many patients are likely to seek a second opinion for serious diagnoses or complicated procedures [8]. Previous surveys estimated that 16–42% of patients seek second opinions [7,9-11]. The second opinion also became an integral part of many health care systems, featuring a competitive marketing benefit for attracting patients to particular insurance packages.

The search for a second opinion reflects the patient’s desire to obtain the best possible medical treatment, and it may also express the patient’s dissatisfaction with medical treatment or communication with the physician. Second opinion research has focused mainly on the diagnostic discrepancies between independent opinions [4,5,12-15], the reasons for which patients seek second opinions [16,17], and the characteristics of these patients [10,18,19]. Yet data on the physicians’ perspective are scarce [20]. Because physicians have a key role in these consultations, it is important to explore their perspectives on second opinion. As such, they profoundly affect how the practice is utilized, the dynamics with the patients, and policies related to second opinion.

### Second opinion in the Israeli healthcare system

The Israeli healthcare delivery system consists of four health funds that are public organizations, providing primary and secondary care. Tertiary care is provided by government-owned hospitals, public hospitals not owned by the government, and private for-profit hospitals. In parallel, private health services are provided by physicians seeing patients in private clinics. For several reasons, it is very common that physicians work across settings, e.g., at both a public hospital and a private clinic.

Patients in Israel are entitled “to obtain another opinion on the matter of treatment” by the Israel Patient’s Rights Law. Nevertheless, there is no specific mechanism, including financial cover, for this entitlement in the National Health Insurance Law. Practically, patients in Israel can obtain second opinions in two ways:

(1) **Through the secondary care provided by the health funds**, by specialists working in the community and outpatient clinics in hospitals. The health funds in Israel permit elective direct self-referrals to at least five specialties (obstetrics and gynecology; ophthalmology; ear, nose, and throat; dermatology; and orthopedics). Referrals to other specialties must originate from a GP or another physician, but this is not the case for all health funds. The patient co-payment for visiting a specialist in community secondary care is equivalent to $6 per specialty, which covers an unlimited number of visits (from the date of payment until the end of the quarter), but all the visits must be with the same specialist. Should patients wish to consult with another specialist in the same specialty, they have to wait for the quarter to end.

(2) **Through the private sector.** Patients can get a second opinion through the private sector and pay completely out-of-pocket. Alternatively, patients enrolled in the health funds’ supplementary health insurance programs are eligible for partial reimbursement for out-of-pocket second opinion consultations. According to the National Health Insurance Law, the health funds are allowed to offer their insurants supplementary health insurance programs in addition to what is covered by the basic basket of health services stipulated by the National Health Insurance Law. The supplemental health insurance programs are designed to extend coverage by providing reduced co-payments as well as choice of physicians and hospitals, yet the direct out-of-pocket payment is considered to increase health disparities. These programs are voluntary and their policies vary among the various health funds. According to the Ministry of Health, 73.1% of the people insured by the health funds had supplementary health insurance in 2010 [21]. Patients with supplementary health insurance are entitled to obtain a second opinion in the private sector and to be reimbursed for 80% of the out-of-pocket cost, limited to an equivalent of $130 per consultation, up to three annual consultations with no quarterly restrictions. This coverage is fairly similar across the four health funds. The out-of-pocket cost of a private consultation is roughly equivalent to $200–500. Hence the reimbursement that patients receive from the health funds for second opinions is only partial, thus making second opinion still quite costly, especially for those who are in lower socio-economic classes of Israeli society. Yet it is important to clarify that private consultations are not limited to second opinions, and the latter may not even constitute a large proportion of private consultations; patients may seek private consultations for other purposes than a second opinion.

Hence, in practice, the health funds, belonging to the public medical sector, subsidize second opinions through both community secondary care and the supplementary health insurance programs. The health funds’ total net expenditure on second opinions reimbursed through the supplementary health insurance programs in 2010 was equivalent to $86 million (after subtracting co-payments) [21]. This is the second largest expenditure after surgery, accounting for 14% of the health funds’ net expenditure on services consumed through the supplementary health insurance programs. Patients in Israel can also purchase supplementary insurance from commercial health insurance companies that also cover second opinions.

Because second opinions are commonly provided by private consultants, it is important to briefly describe how the private system works. In Israel, it is relatively easy for physicians to combine private and public practice. Most physicians work across settings, and very few physicians work only in private settings. Yet there are no clear mechanisms for physicians to combine private and public practice, to move from public to private or vice versa, and no clear regulations regarding this practice. There are also differences among the various health funds and hospitals. To the best of our knowledge, there are no data about the differences between physicians who work in a private setting and those who remain in the public sector. A recent study has shown that physicians practicing in the center of Israel have many more opportunities to practice private medicine than those practicing in the periphery [22].

Only a few studies referred indirectly to second opinion in Israel, discussing issues such as the barriers to health resource allocation [23], patient visits in secondary care [24], ambulatory services utilization [25], and elective surgery [26]. Second opinion is quite common, at least in some specialties: A survey of 103 cancer patients showed that although 88% of them reported that they relied on their oncologist for therapeutic decision making, 45% indicated that they had sought a second opinion [27]. In a recent survey of 332 Israeli physicians, we showed that the judgment of physicians giving second opinions was in some cases affected by other physicians’ opinions, but unaffected in other cases [28]. Our goal was to explore the attitudes of Israeli physicians toward the second opinion, ultimately to devise potential policy recommendations for the efficient use of the second opinion.

## Methods

### Design

The study was part of a larger mixed methods study aimed at exploring various aspects of the second opinion. Another part of the study aimed to evaluate whether physicians’ decision-making is affected by the patient having obtained another opinion. In parallel to a quantitative survey described elsewhere [28], we conducted a qualitative study based on personal interviews to probe more personal dimensions of second opinions. Qualitative methods enable subtexts, attitudes, and motives to be explored [29]. A detailed description of the method appears elsewhere [30]. We designed a narrative-phenomenological study. Phenomenological research aims to describe an experience of a phenomenon. The data for this kind of qualitative analysis are narratives, i.e., using field texts as data sources (stories, autobiography, journals, etc.). This is a suitable method for the goal of the study, because it enables exploration of the experience, attitudes, and belief that physicians have about second opinion from their daily clinical work. We conducted semi-structured, personal interviews with physicians. Such an interview is systematic while remaining sensitive to the dynamics of the conversation, enabling each interview to evolve according to the themes that are brought by the interviewee. The interview focused on the beliefs, attitudes, feelings, and behaviors that emerge in second opinion encounters (see Appendix A). We asked questions about implementation of the second opinion, daily experience with second opinions, patients’ motivation for seeking second opinions, physician-patient dynamics, and questions related to policy and implementation. The initial questions came from the literature on second opinion. The protocol was tested with two senior peers, evolved during the first interviews, and stabilized after the fifth interview.

### Participants

We interviewed a convenience sample of 35 orthopedic surgeons and neurologists in Israel, all of them specialists who provide second opinions. These specialties were chosen because they involve high rates of second opinions and they enabled us to compare a surgical specialty to a non-surgical one. The eligibility criterion was being a specialist who provides second opinions. The sample included senior residents (more than 6 years of residency), junior specialists (up to 7 years of post-training experience), and senior specialists (more than 7 years of post-training experience). We produced lists of possible consultants from Internet sites of orthopedic and neurology wards. Other consultants were approached through peers. The entire population in Israel includes 375 orthopedic surgeons and 250 neurologists. We contacted 52 physicians, of whom 35 were actually interviewed (response rate 67.3%). The protocol was approved by the Institutional Ethics and Human Subjects Review Committee of Soroka University Medical Center and by the Ministry of Health Department of Clinical Trials. Participation was voluntary and the participants were not provided with any incentive.

### Data collection

The interviews were conducted in the clinicians’ offices from January 2007 to July 2008 by researchers (GG, ND) trained in gathering and analyzing qualitative data. Interviewee anonymity was ensured while interviews were recorded and transcribed.

### Analysis

We used the Grounded Theory approach, where codes and categories emerge from the data without preconceived expectations [29]. We conducted a hermeneutic analysis, in which we iteratively coded the data and categorized the codes into themes. Two researchers (GG, ND) independently analyzed the interviews and conducted ongoing discussion sessions with the other researchers. The data were analyzed using ATLAS® software. The final themes are the outcome of an iterative definition process.

## Results

The sample characteristics are described below and also detailed elsewhere [30]. In total, 20 neurologists and 15 orthopedic surgeons participated in the interviews. Most of the participants were senior specialists (51.4%, 9 neurologists and 9 orthopedic surgeons), or junior specialists (40.0%, 10 neurologists and 4 orthopedic surgeons). We also interviewed 3 senior residents (1 neurologist and 2 orthopedic surgeons). Most of the participants were male (91.4%, 17 neurologists and all the orthopedic surgeons). Most of the participants obtained their medical education in Israel (65.7%, 11 neurologists and 12 orthopedic surgeons). The main setting of practice of most of the participants was a public academic hospital (91.4%, 17 neurologists and all the orthopedic surgeons). We also interviewed two neurologists practicing in a community specialist service and one neurologist in a private clinic. Most of the participants were practicing in the periphery of Israel, i.e., outside the major cities of Tel-Aviv, Jerusalem, and Haifa (65.7%, 15 neurologists and 8 orthopedic surgeons). Most of the participants were providing second opinions in private settings (60.0%, 10 neurologists and 11 orthopedic surgeons) in addition to their public work. In both settings, they invest a different amount of time in giving second opinions; while for some it is a part of their other duties, for others (mainly in the private setting) this is their major work.

Most of the physicians expressed a positive attitude toward the second opinion. Beyond being a patient’s right, they describe it as a legitimate and often justified tool that can improve overall quality of care. They felt that when used properly, the second opinion can benefit everyone involved: the patient (by either ratification of the first opinion or by bringing to the patient’s attention additional options he/she should consider); and the physician (by helping the patient make a decision, discussing the patient’s case with the other physician, and as a source of added income). Some physicians even encourage their patients to seek second opinions.

Through our analysis, we identified the players who participate in second opinion consultations (the patient, the two consultants, the insurer, and non-clinical advisors) and their interactions—the patient interacts first with a primary consultant to get a first opinion and then with another consultant for a second opinion. The second opinion consultation takes place in a particular setting (public hospital, health fund, or private clinic) and in a specific geographical area. These consultations are provided or reimbursed by an insurer or the patient. Sometimes patients seek advice from a rabbinical medical broker outside the healthcare system. Using these definitions and contexts, we arranged the data along several themes: second opinion in public vs. private medicine; second opinion in the center of Israel vs. in the periphery; utilization of second opinion; and medicine between science and religion. We discuss these themes below.

Codes in square brackets (e.g., [N9]) are interviewee identifications, where [N] represents a neurologist, and [O] represents an orthopedic surgeon. Direct quotations from the physicians were translated from Hebrew with particular emphasis on preserving the original meaning and tone of the physicians’ remarks.

### Second opinion in public vs. private medicine

The way second opinion is being provided (described above) may create tensions between the settings of public and private medical care. According to the physicians interviewed, patients obtain their second opinions mainly in the private sector [e.g., O3, O4, O6, O7, O9, O11, O15]. According to the physicians, most patients either see a community consultant in their health fund, or get a reimbursement from their health fund via the mechanism described in the Introduction. The latter is more costly to the patient and hence patients expect much more in terms of length of visit and personal attention to their case.

Second opinion has a different meaning in the different settings: The physicians made a strict distinction between an “opinion” provided as an integral part of the diagnosis and consultation during the natural course of being an inpatient. A “second opinion” is another opinion that the patient seeks outside of the hospital. While most of the physicians were supportive of second opinions outside of the hospital, many of them stated that providing a second opinion within the hospital is forbidden, and some even referred to it as a “collegial taboo” or even a “crime” [N2]. The physicians practicing in community consultation care (secondary care) viewed the second opinion of a colleague practicing in the same clinic as a legitimate act, or at least were not affronted by it.

#### **Patients’ reasons for seeking second opinions in the private market**

Physicians mentioned several reasons for patient preferences, as the physicians perceived them, for a private physician when seeking a second opinion.

Belief in the superiority of private consultation

According to the physicians, most patients have a tendency to overestimate the quality of a private consultation and, therefore, they perceive a private medical opinion to be superior to that given in a public setting. Although there is a rationale to support this belief, as physicians practicing in the private market are usually more senior, some physicians mentioned that this perception may mislead the patient. A second opinion from a senior physician in a public setting may be better than that from a less experienced physician in a private setting. Moreover, physicians interviewed mentioned what they perceive as the patients' lack of credible information about the quality of privately practicing physicians.

Personal selection of a consultant

An essential component of the second opinion consultation is the element of patient choice [O4, O9]:

"*“… You go to a doctor that YOU choose, unlike the public system where you get the one who was just available … it gives the feeling that they choose one that they really want, after research and recommendations … so they feel more confident and are more inclined to accept the consultant’s opinion …” [O4].*"

For example, one physician [O5] recalled a trial program to provide second opinions as a private service within a public hospital by senior physicians practicing in the hospital. This effort failed, however, because patients wanted to choose the second opinion consultant themselves, an option that was not available in this trial.

“Personal time”

According to the physicians, many patients are motivated to seek a second opinion for emotional reasons, such as anxiety, and by a desire to get more information and relief. The usual hectic public setting allows very limited time to spend with each patient. But in the private setting, the physician can spend the necessary time with each patient, have a relaxed conversation, and discuss diagnosis, treatment, and prognosis [O15]. The patient effectively “buys the physician’s time” [O2] and the consultant can spend more time evaluating each case, discussing patient preferences, considering further studies, and explaining the diagnosis, treatment, prognosis, etc.

"*“… Sometimes a visit in a public setting lasts too long because there are too many questions, and you might become impatient, and the patient also feels that. So the second opinion provides another opportunity to get more information, perhaps about other nuances, I think that is positive …” [O11].*"

“I’ve done everything I could”

Obtaining a second opinion is often a part of how the patients cope emotionally with their disease. The high costs of private second opinions may constitute part of the “I’ve done everything I could” feelings of the patient.

#### ***Physicians’ attitudes toward the private second opinion***

The physicians expressed ambivalent attitudes toward the private second opinion. While some proclaimed it a necessity, others noted what they considered its problematic aspects:

"*“… Second opinion, essentially, is an example where private medicine celebrates the current limitations of public medicine, and perhaps, the failure of public medicine. People can’t find satisfactory solutions, so they turn to private medicine …” [O9].*"

Several physicians talked about their feelings of unfairness triggered by the huge efforts they often invested in the public settings and the disproportionately small rewards they receive relative to physicians in the private settings [N6, O5]. For example:

"*“… In the public sector, it often happens that you are taking care of a patient for two weeks, doing screenings, imaging, weekend duty,* etc.*, and then the patient goes to someone else, who sits with the patient for 30 minutes, reads what you have sweated for, and takes a thousand shekels, and you’ve worked here for free, it’s annoying, it’s frustrating …” [N6].*"

#### ***Maintaining medical ethics in the private second opinion***

The physicians also mentioned the challenge of reconciling the tension between clinical and economic considerations and of maintaining medical ethics regardless of the setting. Some physicians felt that unlike the public setting, in a private setting the physician is driven by a financial motive to satisfy the patient [O6, O15]. However, most physicians said that they maintain the same clinical judgment whether the setting is public or private, and they uphold professional ethics by trying to avoid financial considerations [O1, O2, O4, O5, O13, O15, N7].

"*“… The whole idea is to try and stay straight, and not suggest surgery just because of money … “[O15]*"

#### ***Private second opinion in hospital settings***

Several public hospitals provide private services, including second opinions. Hadassah Hospital, for example, is a public not-for-profit hospital, not owned by the government, which provides private consultations. The reimbursement mechanism for getting a second opinion in these services is similar for other consultations with private physicians (i.e., 80% of cost, up to approx. 550 NIS, up to 3 annual consultations). Some physicians (e.g., [O15]) mentioned the prestige of these hospitals relative to “regular” private consultations in a non-institutional setting. Another physician referred to a trial to establish such a service in a peripheral hospital (i.e., private consultation service within a public hospital) that was unsuccessful because the patients realized that the physicians were the same physicians practicing in the public hospital. Hence, we assume that second opinions provided in the private service of the public/private not-for-profit hospitals pose the same kind of issues and problems as second opinions provided outside the confines of these institutions.

In addition, the setting may affect the way second opinion is practiced. Hospitalized patients are less inclined to seek a second opinion because they feel that within the framework of normal hospital procedures, several physicians discuss their case [O11]. Some hospitalized patients invite physicians from outside the hospital to give them second opinions in the ward, but this is relatively rare [O5, O15]. It may also affect the patient-physician relationship:

"Interviewer: “*Is it common that patients hear your opinion and then seek a second opinion from someone else?*”"

"Interviewee: “*Frankly, in all of those cases I initiated the second opinion; I cannot recall anyone who heard my opinion, and went to someone else and then came back to me, or that I heard they went to someone else. I work in a private, very exclusive milieu. I guess that the setting plays an important role*” [O14]."

### Second opinion in the center of Israel vs. the periphery

One of the most common expressions, made mainly by physicians practicing in the periphery, is the patient perception that “the phenomenon of the second opinion is routine in the ‘center’” (i.e., Tel-Aviv metropolitan area and hospitals in Jerusalem) and that “the best physicians are in the center” [N2]. According to this perspective, patients consider physicians practicing in central regions of Israel as more knowledgeable and of greater expertise than those in the periphery, and, therefore, it is important for patients to meet “the expert from the center”, even when such physicians possess the same training and experience as a physician practicing in the periphery. Going to “the doctor from the center” instills in patients the confidence and the feeling that they did everything they could to find the best treatment [O6]. Patients from the periphery come to the center for a second opinion, but seldom the other way around. A common feeling expressed by physicians practicing in the periphery is that patients devalue their expertise and perceive them as inherently under-qualified compared to the physicians practicing in the center. One physician described an ironic situation, in which physicians who work in the periphery are more highly esteemed by their patients if they also have private clinics in the center [N2]. The following is another anecdotal example of patient over-evaluation of physicians who practice in the center:

"*“… I had a patient whom I advised that she needed surgery, but then she was gone and returned after a month. Apparently she waited for a whole month for a very famous doctor in the center, just to hear him say that I’m right, and that I’m the best surgeon for it. That’s fine, but she waited a whole month, and meanwhile the fracture deteriorated, the risk increased and eventually she was not operated. She spent a month which was very, very critical, and screwed her chances, only to hear a very famous person …” [O15].*"

According to the physicians, several structural reasons can be cited for the low utilization of the second opinion by patients in the periphery. For example, in the periphery 1) there are fewer experts and hence, less choice for patients; 2) private medical care is less developed, especially for private surgery; and 3) supply of and demand for private medicine are much lower and fewer people have adopted the mentality that accepts and promotes using private medicine [N2]. Patients living in the center are usually more aware of the option of getting a second opinion, and even those from the low-medium socioeconomic classes in central Israel obtain second opinions. Moreover, they are often more aware of why the second opinion is important [N2, O2]. Although such inequalities exist throughout Israel, they are more prominent in the peripheral regions where patients of limited means can be found in greater numbers, and therefore, many physicians prefer to work in the central regions of Israel. Physicians who practice in the periphery are often frustrated by their perception of being under-valued by patients and generally unappreciated merely because they practice in the periphery:

"*“… The assumption of most patients is that when working in the periphery, you are professionally inferior to doctors doing the same work in the center. It is annoying, because it’s the premise of most of the patients, and if it is their reason to hear a second opinion, it’s annoying … I mainly face it with patients whom I offer something and they turn to doctors in the center, because they think they are better, because they are ‘doctors in the center’ … I say this with sad cynicism” [O15].*"

According to the physicians interviewed, there are legitimate reasons to consult physicians practicing in the center: because some services are supplied better by central hospitals than by those in the periphery [O15] and because different hospitals also have different approaches to the same treatment [N2]; the second opinion consultation is legitimate and even useful. However, the physicians have expressed frustration from being automatically devalued relative to the physicians practicing in the center of the country.

### Utilization of second opinion

Opinions among physicians were mixed about the level of second opinion utilization. Several neurologists stated that there has been an increase in its use but that many patients are still unaware of the existence and/or importance of this tool, which is sometimes used inappropriately [N4, N5, N6, N8, N9]. Others stated that many patients seek second opinions [N14, N15], and some orthopedic surgeons pointed to an overuse of second opinion consultations due both to patient anxiety and to such consultations being too accessible, thus leading to many unnecessary consultations for relatively simple problems [O2, O10, O11, O12]. Some physicians mentioned the need to increase awareness and to reduce co-payments to encourage second opinion utilization [O5, O7, O8].

Most physicians agreed that the second opinion has been increasingly utilized in recent years due to the reimbursement that patients now receive from their health funds. The health funds’ policy regarding second opinions has increased the legitimacy of such consultations and made them more accessible. The physicians also mentioned that the increased availability of medical information on the Internet and in the media has made patients more knowledgeable about their diseases and more skeptical of their physicians. Some physicians referred to the trend of legal claims against physicians as contributing to the increase in second opinion utilization, ultimately leading to unnecessary and redundant second opinion consultations and the phenomenon of “doctor-shopping” (i.e., getting many opinions for the same episode), which may be the result of the lack of a mechanism that helps patients to reconcile discrepant opinions [O1, N10].

Although physicians mentioned that it is difficult to characterize the patients who seek second opinions, some mentioned anxious patients as those with the greatest tendency to request additional consultations about their health. Moreover, the patient asking for a second opinion is typically from among the more educated sector of the population, of high socio-economic standing, with access to sources of information, and the ability to pay for a second opinion [O2, O4, O13, O14].

### Medicine between science and religion

According to the physicians, many patients in Israel get rabbinical consultations, in parallel with their clinical consultations, about surgery or treatment regarding selection of the hospital or physician [O2, O3, O4, O11, O14, O15, N1, N6, N8]. This practice is used mostly by ultra-orthodox Jewish people, but for different reasons some members of the secular public also consult Rabbis about medical treatment. In the ultra-orthodox community, the rabbinical consultation is an integral part of getting a Rabbi’s blessing (i.e., approval) for a medical procedure. The Rabbi usually directs the patient to a specific specialist or hospital. Secular patients, on the other hand, seek advice from rabbinical medical brokers to get additional opinions based on their sources and contacts.

But physicians are not wholly satisfied with rabbinical involvement, as it casts doubt on their professional judgment, and it creates tension between “faith” and “science”. The physicians were also uncomfortable with the patients’ need to receive approval from a Rabbi after the physician recommended a decision and being “dictated” to by a Rabbi, which contradicts the perception of the professional authority and clinical autonomy of the physicians. Some physicians expressed some distrust of Rabbis as a source of clinical judgment, because they rely on faith rather than on medicine as a scientific profession [N6, O2, O4, O11, O14, O15]. The fact that Rabbis advise patients to be treated in certain hospitals or by certain physicians arouses resentment among physicians [O15]. They also mentioned that ultra-orthodox patients tend to express their desire for a second opinion more blatantly than secular patients, probably due to their perception of the physician as inferior to the Rabbi.

"*“… We have quite a bit of experience here with orthodox people. The decision may be easier for them because they do not decide, but the Rabbi, so it is a kind of a second opinion. Rabbis have their knowledge, but if the patient delivers incorrect information, then their decision may be wrong, but it is sacred. Sometimes the Rabbi objects to surgery although I recommend one. So sometimes it can be problematic in terms of the therapeutic approach” [O11].*"

"*“… In the orthodox sector it is sometimes an integral part of getting the blessing of the Rabbi, and to go to whom the Rabbi ordered you to go. They come from relatively lower socioeconomic levels, but yet they are willing to pay to consult with the physicians to whom the Rabbi sent them” [O14].*"

## Discussion

Our goal in this study was to explore the attitudes of Israeli physicians toward second opinions, through personal interviews. From a sociological perspective, second opinion is a commonly used tool in the Israeli healthcare system, and as such, it represents the prominent tensions apparent in other health services. The players involved in second opinion consultations—the patients, the consultants, the insurers, and non-clinical advisors—have different and sometimes contradictory motives, a reality that can generate conflict. Reconciling these conflicts is among the major challenges behind facilitating the delivery of the second opinion in a respectful, informed, balanced, and accessible manner. We identified four issues in the data: (1) inequalities between central and peripheral regions of Israel; (2) inequalities between private and public settings; (3) an implementation gap between the right to a second opinion and whether it is covered by the National Health Insurance Law; and (4) tension between the authorities of physicians and religious leaders. These issues apparently interact with each other: supply and demand of public and private medicine vary between the country’s center and its periphery, and these differences, in turn, create inequalities that affect the implementation gap. These tensions also operate under macro conditions such as legislation, health policy, culture, and norms. While these tensions are also apparent within the framework of the first opinion, they become more critical in second opinion consultations, which tend to be more complex, require specific sub-specialties, involve more elaborate decisions, and cost considerably more.

### Setting: Second opinion and public vs. private medicine

#### Patient preferences for private second opinions

The tension between public and private medicine is evident in the current struggle over the existence and quality of public health care in Israel as expressed in the recent physicians’ and nurses’ strikes as well as in the Ministry of Health goals to reduce health disparities in Israel. For example, the provision of private medicine in public hospitals in Israel has been a subject of a major debate [31-33]. According to the physicians interviewed, they think that patients who seek second opinions do so in the private sector. Getting a private second opinion apparently has an element of a premium product, with increased individual attention, shorter waiting time, and greater privacy, that is absent from the public system. The physicians interviewed perceived that patients prefer a private second opinion because they believe that “private” physicians are superior to “public” physicians, and likewise, they prefer to choose a consultant by themselves and to have “personal time” dedicated exclusively to them. This is probably one of the reasons for the tendency of patients to attribute greater value to the second rather than to the first opinion [34-36]. Getting a second opinion in the private medical sector also allows patient access to high-ranking professors who, although they serve as department chairs in the public health system, are not easily accessible for patients through public health system channels. Finally, paying for a costly private consultation also helps promote the patient’s feeling that “I’ve done everything I could.”

#### Flaws of the private second opinion

Despite these benefits, such an arrangement has some flaws. First, the private medical sector lacks the same regulatory mechanisms as in the public sector, a situation that may lead to unnecessary and costly second opinions. In a private market, the second opinion becomes a “commodity” that distinguishes between those who can and cannot afford it. Second, the patients’ efforts to choose the “best consultant” under the assumption that “private” physicians are superior to “public” physicians can sometimes be undermined by their lack of valid data on physician quality and performance. Hence, they are prone to rely on the physician’s impression, charisma, and public appeal, and rumors and limited-value data. Surprisingly, although many physicians in Israel work in both the public and private settings, physicians themselves echoed patient assumptions about the professional superiority of private physicians compared to public ones. Yet, one may argue that without current data on the quality of private specialists’ outcomes, the choice of “private” doctors is as informed as a choice of any other physician, including the public sector. However, there are two inherent differences between a choice of a public or private physician. In the public service, patients have less choice of physician, and mostly see an available physician on a first-come, first-served basis. In the private sector, patients proactively seek a physician of their choice. Also, private consultations are costlier than public ones, and hence there is great importance for the patient to choose the best physician they can afford.

What are the implications of publicly employed physicians seeing patients privately for second opinion purposes? First, would the same physician provide a “better” opinion in a private setting? Apparently not. The physicians said that they certainly would not intentionally offer sub-standard care to their public patients. However, the private consultation enables both the patient and the physician more time to discuss the case in a relaxed and informal atmosphere, in which the physician can dedicate adequate time to discuss the history of the case, different diagnoses, and treatment alternatives with the patient. Hence the physician can have a broader view on the case, consider previous diagnoses given to the patient, and hence potentially provide a better decision. But, then, this option is open only to patients who can pay for it. Second, would the same physician provide different opinions in different settings, due to financial interests? The physicians denied such an influence, although some of them mentioned that some cases might fall within a grey area. Some physicians mentioned the challenge of maintaining the same level of professional judgment in the public and private settings. For example, in private settings the physician is usually more interested in satisfying the patient.

While our interviewees argued that the private setting is better suited for second opinion policies, they were concerned about the potential for tension between the public and private settings. For example, several physicians complained about disparate efforts and rewards in the public vs. the private settings. It is important to mention that the physicians did not directly attack patients’ decisions to pursue second opinions from “private” physicians, but the structural issues that cause system failures, such as misinformation about the quality of private physicians, inequality in accessibility, and physicians’ greediness in providing costly second opinions in an unregulated market.

### Location: Second opinion and the center vs. the periphery

One of the major concerns expressed by the physicians, especially by those practicing in the periphery, is the notion that “second opinion is better in the ‘center’ of the country”. Both patients health-seeking behavior and reasons connected with health system structure have led to low patient utilization of second opinion in the periphery relative to the center. This low utilization is related to the fact that patients in the periphery tend to have lower incomes. Nevertheless, physicians practicing in the periphery felt under-valued by patients. Indeed, a recent study has shown that physicians practicing in the center of Israel have many more opportunities to practice private medicine than those practicing in the periphery [22]. The conditions generate unequal access, for both patients and physicians, to second opinion consultations. While inequalities in Israeli health services are a known fact [37], differential access to second opinion is another, less discussed symptom of these inequalities.

### Implementation gap: The right to second opinion and actual patient coverage

Such a structural gap between an existing right and its practical implementation could create inequalities in accessibility across different populations and geographical regions. While most physicians stated that utilization of the right to a second opinion has increased in recent years, there were mixed opinions about the frequency of second opinions (i.e., how many patients in the practice seek second opinions) and appropriateness (i.e., how many of these second opinions are indeed justified). Although some physicians claimed that the option for a second opinion has been underused, others felt that it suffered from overuse or even misuse. Indeed, a literature review by the authors [20] found a lack of data about the level at which second opinion is utilized in Israel.

### Authority: Second opinion between science and religion

Another common form of second opinions in Israel, though not purely medical, is a consultation with a rabbinical medical broker in parallel with the clinical consultation. The ultra-orthodox community, as well as secular patients, turn to religious authorities because of their vast "local" knowledge of which physicians are best for which problems, and their ability to sometimes speed access to these physicians. This information and referral function is separate from the issue of giving blessings, and is a practical, rational response to the lamentable lack of clear information for patients about which physicians are best, in either the private or public sectors.

Most of the physicians were dissatisfied with rabbinical involvement because they were concerned that patients are being misled by unprofessional judgment. They were also concerned that it casts doubt on their professional clinical judgment and engenders tension between faith and science, as physicians feel devalued by Rabbis. In a previous study, a third of 103 cancer patients surveyed reported seeking the opinion of a rabbinical medical broker [27]. Another survey with Israeli physicians reported that they respect Rabbis’ suggestions in the area of medical decision making though they would not let a Rabbi’s advice interfere with their decisions if they believed the Rabbi’s opinion went against medical opinion [38]. Yet it is unclear how these two mechanisms should be integrated and even regulated in the context of the second opinion.

### Limitations

The qualitative methodology applied in this study can identify issues, features, attitudes, motives, and barriers within the context of the second opinion. Yet our study carries some limitations. First, qualitative research is often critiqued as subjective, but the systematic approach to data gathering and data analysis, as well as establishing detailed records of the analysis, counters this concern. Second, most participants were hospital physicians whose perspective may over-represent this setting. Yet due to the structure of the healthcare system, almost all of them provide second opinions in the community and/or private settings. Hence, although they were physically interviewed in a hospital, their perspective also reflects their experiences in other settings. Our study focused on physicians' perspectives and should be complemented with interviews with patients in order to have a comprehensive picture on second opinions.

### Possible policy implications

The findings can serve to derive potential policy recommendations by highlighting areas needing structural changes and further investigation:

#### Regulation and structure

Currently patients have large out-of-pocket costs on second opinions, and the health funds spend very heavily on second opinions through their supplementary health insurance programs. The right to receive a second opinion raises the issue of how a public system should provide at least some of the perceived benefits of the private system, thereby decreasing inequalities for patients and physicians alike. Can the public system provide high-quality, accessible, objective, and affordable second opinions? Can the public system potentially provide the benefits of private consultations, such as adequate doctor-patient time, patient selection of consultants, and patient accessibility to high-ranking professors? If a public system was ideally able to provide conditions similar to those in a private clinic, would that decrease the demand for private second opinions and facilitate equality? While there is currently no clear answer to these questions, the increasing demand described by the physicians for private second opinions reflects the general privatization trend that healthcare services in Israel have been undergoing. Based on a vast body of research that showed how second opinion can change the course of treatment [4-6], we suggest that the second opinion should be institutionalized and regulated to ensure equality and fairness. A better solution would probably be to provide the public system with mechanisms and resources to provide better care, rather than let the privatization trend reinforce the drawbacks of the public system. One option is to provide second opinion as a public service by a designated “second opinion clinic” or other innovative mechanisms such as polyclinics [39]. A polyclinic is a clinic where key providers such as family practitioners, consultants, social workers, and other allied health professions are located under one roof, enabling enhanced collaboration among services. Policies are required to promote the optimization of treatment and the avoidance of unnecessary risks, and they will help assure the quality of treatment while saving costs by preventing unnecessary treatments.

#### Strengthening doctor-patient dialogue on second opinion

Both the public and the medical profession should be informed on patients' right to second opinion, and should be directed on how to utilize second opinion to benefit from it as much as possible. Underuse can be treated through education, improved accessibility in peripheral regions, and co-payment reductions. Overuse (i.e., “doctor-shopping”) is sometimes the result of choosing an inappropriate consultant for a second opinion and the lack of a mechanism to help patients reconcile discrepant opinions. Hence, mechanisms for guiding both physicians and patients to an appropriate mechanism to implement the second opinion process, and for making an informed choice between the two opinions, can be implemented in parallel with control over utilization.

#### Further investigation

There is a need for studies that will examine the prevalence of second opinions overall, as well as by specialty and by various patient characteristics. This has importance for further understanding the overall nature and significance of the second opinion phenomenon, and in particular for exploring the extent of inequalities. In addition, decisions about whether to add second opinions to the national health basket would benefit from the results of cost-effectiveness analyses. Finally, further research is needed to complement physicians' perspectives with a better understanding of how patients perceive second opinions and to learn how significant the gap is between the right and its implementation, so as to inform sound implementation recommendations.

## Conclusions

Second opinion is a complex tool that requires further study to be optimally implemented. The take-home message of this article is that all the physicians agreed on the importance of the second opinion as a clinical tool, but raised concerns about the way it is provided and utilized. Meanwhile, the second opinion reflects the predominant tensions in the Israeli healthcare system, mainly inequalities between public and private medicine, and inequalities between central and peripheral regions. The National Health Insurance Law should strive to provide the option for a second opinion as stated by the Patient’s Rights Law. Further research is needed to complement physicians' perspectives with a better understanding of how patients perceive second opinions and to learn how significant the gap is between the right and its implementation, to advise sound implementation recommendations.

## Appendix A. Interview protocol

### Adapted from Greenfield et al. [30]

Hello, my name is…. This interview is a part of a study conducted at Ben-Gurion University of the Negev about physicians’ views on second medical opinions. The interview will be used for research purposes only and is anonymous. The interview will take about 30 minutes and will be audio-taped with your permission. Thank you in advance for your cooperation.

### **“**Warm-up**”** questions

· If you could choose 2–3 words to describe what a “second opinion” is, which words would you choose?

· When you hear the term “second opinion”, what is the first connotation that crosses your mind?

· Please tell me a bit about your interaction with second opinions. Generally, what do you think about second opinions?

### The primary physician’s role: Dealing with patients who wish to seek a second opinion

· How often do your patients seek a second opinion? Do they seek too many second opinions, or too few?

· What, in your perception, triggers patients to seek a second opinion? How can they benefit from it?

· How do patients communicate their desire for a second opinion? Do they tell you about their desire?

· Can you recall a specific patient who sought a second opinion? What was special about this patient? Why do you think that the patient wanted to get a second opinion? Was he/she a difficult patient? How did you feel about it? Were you bothered by the fact that the patient wanted to seek a second opinion? How did you deal with this patient?

· Do you encourage patients to seek a second opinion? Do you initiate it?

· How did you feel when a patient of yours sought a second opinion from someone else, and returned with the second opinion to you? Would you consider adopting the second opinion?

### The second opinion physician’s role: Giving second opinions

· How often do you give second opinions? In which setting?

· If you would go to another physician as a patient, would you tell the physician that you already had a first opinion? Why (why not)?

· Can you please tell me a bit about the dynamics with patients who come to hear a second opinion? Do patients tell you that they already heard a first opinion? How do they communicate with you?

· How would you feel if you eventually realized that the patient concealed the first opinion from you?

· Can you recall a specific patient who came for a second opinion? What was special about this patient? Why do you think that the patient wanted to get a second opinion? Was it a difficult patient? How did you feel about it? How did you deal with this patient?

· To what extent do you feel that the first opinion changed your judgment? Did you employ different considerations compared to the situation where you would have given the first opinion?

### General questions

· There is an argument that using second opinions in an informed manner can decrease the number of legal claims. What do you think about this?

· Does the issue of second opinion arise in informal chats with colleagues?

· How do you compare the way second opinions are practiced in Israel to other countries? Is there a country that can serve as a model?

· (Closing question): Is there is something else that you would like to expand on second opinions, perhaps something we did not discuss?

## Competing interests

The authors declare that they have no competing interests.

## Authors’ contributions

GG was involved with acquisition of funding, conception and design, data collection and analysis, and drafted the manuscript. JSP was involved with acquisition of funding, conception and design, data collection and analysis, and revising the manuscript. SW was involved with conception and design and data analysis, and revising the manuscript. ND was involved with acquisition of funding, conception and design, data collection and analysis, and revising the manuscript. All authors read and approved the final manuscript.

## Author information

**Geva Greenfield, PhD**, is currently a research associate at the Department of Primary Care and Public Health, School of Public Health, Imperial College London. He specializes in health behavior, patient-physician relationship, eHealth, and medical decision-making.

**Joseph S. Pliskin, PhD,** is the Sidney Liswood Professor of Health Care Management at Ben-Gurion University of the Negev. He is a member of the Department of Industrial Engineering and Management and of the Department of Health Systems Management. Prof. Pliskin also has an appointment as an Adjunct Professor at the Department of Health Policy and Management at the Harvard School of Public Health.

**Shlomo Wientroub, MD**, is Chair of the Pediatric Orthopedic Department at Dana Children’s Hospital, Tel-Aviv Sourasky Medical Center, and Incumbent, The Goldberg Family Chair in Pediatric Surgery, Sackler Faculty of Medicine, Tel-Aviv University.

**Nadav Davidovitch, MD, PhD,** is an epidemiologist and public health physician. He is an Associate Professor at the Department of Health Systems Management, and Chair, Center for Health Policy Research of the Negev at the Faculty of Health Sciences, Ben-Gurion University of the Negev. His current research deals with health policy, health inequities, health and immigration, vaccination policy, environmental health, and public health ethics.
